# Hands-On Exploration of Cubes’ Floating and Sinking Benefits Children’s Subsequent Buoyancy Predictions

**DOI:** 10.3389/fpsyg.2020.01665

**Published:** 2020-07-21

**Authors:** Johanna E. van Schaik, Tessa Slim, Rooske K. Franse, Maartje E. J. Raijmakers

**Affiliations:** ^1^Educational Studies, Educational and Family Studies, Vrije Universiteit Amsterdam, Amsterdam, Netherlands; ^2^LEARN! Research Institute, Vrije Universiteit Amsterdam, Amsterdam, Netherlands; ^3^Educational Sciences, Education and Child Studies, Leiden University, Leiden, Netherlands; ^4^Developmental Psychology, Department of Psychology, University of Amsterdam, Amsterdam, Netherlands; ^5^NEMO Science Museum, Amsterdam, Netherlands

**Keywords:** variation, buoyancy, science concepts, inquiry-based learning, category learning

## Abstract

Children accrue experiences with buoyancy on a daily basis, yet research paints a mixed picture of children’s buoyancy knowledge. Whereas children’s predictions and explanations of the floating and the sinking of common objects are often based on a single feature (e.g., mass or facts), children’s predictions of novel cubes reveal solution strategies based on mass and volume integrations. Correspondingly, category learning theory suggests that categories (e.g., floaters vs. sinkers) are easier to identify when items mainly vary from one another in the relevant defining features. For example, a set of cubes only varies in mass and volume and hence density, thereby being able to highlight the deterministic role of density when placed in water. Here we asked how item variation during hands-on exploration affects children’s subsequent predictions and explanations of buoyancy. Kindergarteners and first-, second-, and third-grade children individually explored either a set of 10 systematically varied cubes (i.e., systematic condition; *n* = 95) or a set of 10 common objects (i.e., non-systematic condition; *n* = 96) in a water basin. Next, the children predicted the buoyancy of five new cubes and five new common objects one at a time. Subsequently, the children explained their predictions one subset at a time. The children in the systematic condition were more accurate in their predictions of the test cubes than the children in the non-systematic condition. Latent class regression analyses identified three cube prediction solution strategies. The children in the systematic condition were more likely to use a strategy in which buoyancy decisions were made based on an accurate integration of mass and volume, while the children in the non-systematic condition were more likely to use a strategy in which mass was given more predictive load than volume. A third strategy was characterized by guessing. Latent class analyses of the children’s explanations revealed different explanation strategies, each appealing to several features, but as hypothesized, no clear condition differences were found. The findings indicate that even 5 min of exploration with systematically varied cubes can already help children use an advanced buoyancy prediction strategy. This provides evidence in favor of using category learning theory to inform early science education design.

## Introduction

In many Dutch kindergarten classrooms, 4- to 6-year-old can explore what happens when they place objects in water. Buoyancy lesson plans for such water corners typically suggest offering children a collection of common objects to experiment with (Franse, 2007; Kemmers et al., 2007; Mundkur, 2015; Science NetLinks, 2020). While the use of common objects arguably helps children to relate their classroom investigations to their daily experiences, these objects vary over many features (e.g., texture, rigidity) that are unrelated to their buoyancy. This surface feature variation therefore might come at a cost: it could make the relevant feature (i.e., density^1^) harder to isolate. Here we investigated how variation between items during hands-on exploration influences children’s buoyancy knowledge.

A solid object’s buoyancy is determined by its density, which is its mass divided by its volume, relative to the density of the surrounding fluid. Water’s density is approximately 1, and objects with a density of less than 1 float in water, while objects with a density greater than 1 sink. Density is a fundamental concept throughout the physical sciences (e.g., gases and fluids, weather). Furthermore, density is an example of proportional reasoning, entailing a multiplicative relation between quantities, which, in turn, is central to mathematical and scientific topics (Siegler, 1981; Boyer et al., 2008). Consequently, children’s understanding of buoyancy and density has been the subject of developmental and educational research from multiple perspectives (Piaget and Inhelder, 1974; Smith et al., 1985; Kohn, 1993; Penner and Klahr, 1996; Schneider and Hardy, 2013).

From a very young age, children gain experience with buoyancy in different contexts and for a range of objects. This suggests that they are starting to construct knowledge of buoyancy before necessarily being able to verbalize this knowledge. Following Pine and Messer (1999) we will refer to these different types of knowledge as implicit and explicit knowledge, respectively. In this sense, a child’s explicit knowledge necessitates a conceptual representation (although not necessarily a scientifically accurate one), while her implicit knowledge is not (yet) verbally accessible but can be used to (successfully) perform a task (Karmiloff-Smith, 1992; Pine and Messer, 1999, 2003; Messer et al., 2008). Buoyancy studies indeed indicate that both the nature of children’s knowledge (i.e., implicit vs. explicit) at a given age as well as the types of items used to elicit children’s buoyancy knowledge (i.e., common objects vs. novel cubes) paint conflicting pictures of children’s developing understanding (Rappolt-Schlichtmann et al., 2007; Kloos, 2008; Franse et al., under review).

Children’s predictions of whether common objects will float or sink (which could be made based on either implicit or explicit knowledge) are typically quite accurate. For example, in one study, kindergartners’ and second-graders’ prediction accuracies ranged between 60 and 90% (Rappolt-Schlichtmann et al., 2007). In a study by Franse et al., (under review) 4- to 12-year-old predictions of a boat, coin, ball, and pebble likewise averaged between 88 and 92% accuracy, with predictions significantly improving with age. Children’s predictions were likely accurate because they were making use of known facts or experiences. Correspondingly, the majority of these children’s explanations of why these objects float or sink (which requires explicit knowledge) relied on relevant facts, with mass explanations in second place (Franse et al., under review; see also Smith et al., 1985; Tenenbaum et al., 2004). Thus, young children’s implicit and explicit knowledge in the context of common objects both seem often to be based on one feature (i.e., facts or mass).

Whereas the floating and the sinking of common objects can be learned through daily experiences, this is not the case for novel items. Surprisingly, when young children are presented unfamiliar cubes one at a time, which vary only in mass and volume and, hence, in density, they are relatively accurate in predicting which will float and which will sink (Kohn, 1993; Kloos, 2008). This effect was analyzed further in the aforementioned study by Franse et al. (under review). In this study, children compared items (cubes and common objects) one at a time to two reference cubes (a sinker and a floater) and predicted the item’s buoyancy before subsequently explaining these predictions per set of items. The authors analyzed the way the cubes’ features (e.g., mass and volume) were used by children to decide their buoyancy. The majority of the children’s predictions were shown to utilize a solution strategy that was based on an integration of mass and volume. Children’s use of these solution strategies, and the accuracy of the solution strategies, increased with age; while most 4- to 5-year-old guessed, the majority of children 6 years and older used integrative solution strategies, and the higher the age, the more advanced the solution strategy. Conversely, the same children’s explanations for the buoyancy of the cubes were largely one-dimensional, with the majority of children relying only on mass (Franse et al., under review).

Taken together, across common objects and cubes, prediction accuracy increases with age. However, the predictions of objects are typically based on facts, while (the implicit) predictions of cubes reveal the children’s emerging ability to use the relevant features of mass and volume by the end of kindergarten. Explanations regarding common objects and cubes are both largely one-dimensional and hence inaccurate. Improving (young) children’s concepts of buoyancy would thus require both drawing attention toward the less apparent features of items (e.g., density) and a shift toward multidimensional thinking (Kuhn et al., 2008; Schneider and Hardy, 2013). Considering the integrative solution strategies found when children were simply presented with cubes without experimentation (Franse et al., under review) it would seem that allowing children to experiment with a set of cubes could further trigger children’s attention to the integration of mass and volume (Franse et al., under review).

This proposition finds backing in category learning studies and theory. This research shows not only that humans are quick to identify relations across items by detecting the invariant features but also that such learning is dependent on the category structure (Erickson and Kruschke, 1998; Ashby and Ell, 2001; Sloutsky, 2003, 2010; Kloos, 2008; Kloos and Sloutsky, 2008; Goldwater et al., 2018). Categories that are dense, that is, that have multiple category-relevant features (e.g., shape and behavior of dogs) and few category-irrelevant features (e.g., floppy or upright ears), are easy to learn because of the abundance of defining, invariant features. Sparse categories, however, have multiple irrelevant features and only few relevant, invariant features, making them hard to learn without guidance (Kloos and Sloutsky, 2008). Scientific concepts, such as density, are often based on sparse categories due to their multi-contextuality. Hence, conceptual science learning requires being able to distill the harder-to-detect features across multiple instances. To do so, attention needs to be shifted from irrelevant features (e.g., texture) to relevant features (e.g., density). This selection is challenging and likely requires executive functioning systems (Sloutsky, 2010) which are notoriously prolonged in their development (Diamond, 2013). To facilitate this process, simplifying the learning input (i.e., making the instantiation of the category denser) should make it easier for children to focus on the relevant features simply because there are fewer irrelevant features to focus on. This stands in contrast to what is often done (or advocated) in educational practice. Buoyancy lesson plans often suggest using (a variety of) common objects (Franse, 2007; Kemmers et al., 2007; Mundkur, 2015; Science NetLinks, 2020). The motivation to select a range of objects could derive from a constructivist idea that experiences which build upon prior experiences facilitate knowledge construction (Campbell, 2015).

In summary, findings on children’s knowledge of buoyancy are scattered and depend on how the knowledge was elicited. While children’s predictions about the buoyancy of common objects are typically quite accurate, these are likely based on single features such as mass or facts instead of scientifically correct concepts. Intriguingly, children’s predictions of novel cubes reveal solution strategies based on mass and volume integrations. Correspondingly, category learning theory suggests that when relevant features (e.g., density) are more apparent across items (e.g., cubes), these features are easier to learn. Following from this, we asked how item variation during hands-on experience with buoyancy affects children’s implicit and explicit knowledge.

To this end, kindergarten and first-, second-, and third-grade children explored either a set of 10 systematically varied cubes (i.e., systematic condition) or a set of 10 common objects (i.e., non-systematic condition) in a water basin. Following the exploration, all children were presented with five new cubes and five new common objects one at a time in a randomized order. They were asked to predict the buoyancy of these new items and were subsequently asked for buoyancy explanations for the subsets of these items.

We hypothesized that children in the systematic condition would predict the buoyancy of the new cubes more accurately than children in the non-systematic condition. Particularly, based on the past cube prediction findings (Franse et al., under review), we expected to find that the systematic condition children would be more likely to acquire a solution strategy in which they integrate the mass and the volume of the cubes to make buoyancy decisions and that they do so to a higher degree of accuracy than the children in the non-systematic condition. We did not expect to see notable differences between the two conditions in their predictions of the new common objects because children are generally quite accurate at predicting these (Rappolt-Schlichtmann et al., 2007) and all children could still rely on their past experiences and known facts (Franse et al., under review).

With respect to children’s explanations, this study was more exploratory in nature. Studies investigating children’s understanding of buoyancy and other topics show a discrepancy between children’s predictions, which are typically more advanced, and their explanations, which lag behind (Smith et al., 1985; Butts et al., 1993; Rappolt-Schlichtmann et al., 2007). Given this study’s 5-min hands-on exploration session, it is unlikely that the systematic condition children would be able to directly explicate their experimental findings (Pine and Messer, 1999) let alone transfer this knowledge to common objects. Nevertheless, to assist this process, children in both conditions were offered the same regularly timed prompts to stimulate them to explicitly think about their experiments as well as status overviews to guide their experimentation progress (Lazonder and Harmsen, 2016). As such, children with higher prior knowledge to begin with (i.e., children in higher grades) might show some evidence of explaining the new items’ buoyancies based on their exploration.

## Materials and Methods

### Participants

Kindergartners (grades 1 and 2 in the Netherlands) were recruited at seven primary schools. Of the 151 who participated, the data of 40 (26%) were excluded due to the following reasons: confusing the terms floating and sinking (*n* = 2), corrupt video files (*n* = 4), experimenter errors (*n* = 19), stopping the experiment early (*n* = 3), teacher-reported non-normal development (*n* = 1), not sorting items during the exploration session (*n* = 2), and incorrectly sorting more than two of the items during the exploration session (*n* = 9)^2^. Hence, the data of 111 kindergartners (53 girls; Table 1) were included.

**TABLE 1:** Number and age of the participants per grade and per condition.

**Grade**	**Condition**	**Mean age**	**Minimum age**	**Maximum age**	***n* (*n* girls)**
Kindergarten	Systematic	60.15	49	76	53 (24)
	Non-systematic	66.79	50	82	58 (29)
1st grade	Systematic	81.14	75	93	14 (9)
	Non-systematic	82.50	74	96	14 (5)
2nd grade	Systematic	94.91	83	100	11 (8)
	Non-systematic	95.58	87	103	12 (6)
3rd grade	Systematic	107.94	100	121	17 (9)
	Non-systematic	105.92	101	116	12 (2)

Ninety-three first-, second-, and third-graders (grades 3, 4, and 5 in the Netherlands) were recruited at a science museum. Of these, the data of 13 (14%) were excluded due to corrupt video files (*n* = 12) and parent-reported non-normal development (*n* = 1). This resulted in the inclusion of 80 children tested at the science museum; 28 first graders (14 girls), 23 second graders (14 girls), and 29 third graders (11 girls; Table 1).

The final sample consisted of 191 participants (92 girls). A total of 95 participated in the systematic condition and 96 participated in the non-systematic condition. Informed consent was acquired from the parents before participation. This study was approved by the local social science faculty’s ethics committee (ECPW-2018/193 and ECPW-2018/204).

### Design

#### Materials

**FIGURE 1:**
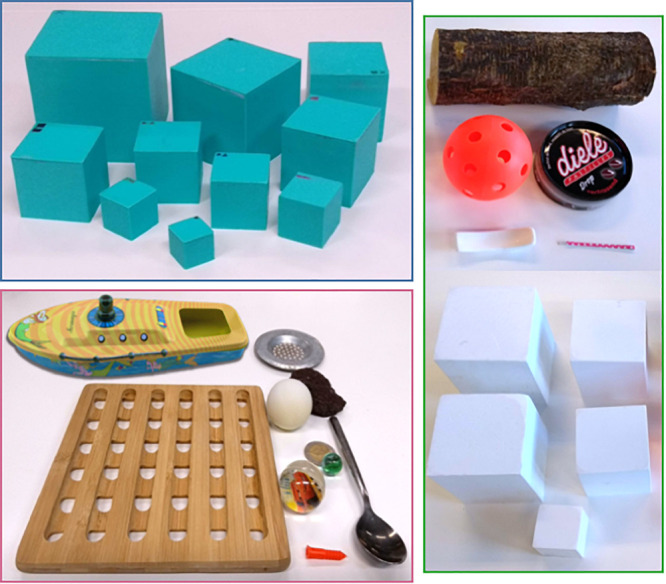
The item sets used in the study. **Top left:** exploration set of 10 cubes used in the systematic condition. **Bottom left:** exploration set of 10 common objects used in the non-systematic condition [boat (F), sink sieve (S), lava rock (S), ping pong ball (F), metal spoon (S), 2-Euro coin (S), marble (S), bouncy ball (F), wall plug (F), and coaster (F)]. **Right:** the 10 test set items used in both conditions (**top:** branch, candy tin, bobby pin, ceramic utensil rest, and floor ball). Note that each cube had a unique symbol (visible in the **top-left** picture) that the experimenter used to identify it, but there was no discernable pattern so that children could not use these to decipher a cube’s buoyancy.

Figure 1 depicts the items used in this study (see also Supplementary Table S1). The items were selected based on past findings and pilot study results. The cubes were made by 3D-printing hollow boxes and lids. These were subsequently filled with Styrofoam, lead pellets, and glue to achieve the desired masses, and the lids were super-glued shut.

#### Pilot Study

For the pilot study, a broad range of items were selected, drawing inspiration from previous studies and buoyancy lesson plans (Franse, 2007; Kemmers et al., 2007; Mundkur, 2015; Science NetLinks, 2020). Forty-three children between the ages of 4 and 10 years were tested individually at a science museum after receiving a signed informed consent from their accompanying guardian. Following an introduction with a water basin to ensure familiarity with the terms “floating” and “sinking,” the experimenter handed items one at a time to the child and asked her to predict whether it would float or sink. Based on these data, a final selection was made, and for the objects, the materials, the shapes, and the presence of holes and hollow spaces were taken into account to create a diverse range. Several items that did not achieve the desired accuracy rates were replaced based on the accuracies of the other items and past studies’ findings; hence, not all items in the final design were piloted. Where available, pilot or previous study prediction accuracy is included in Supplementary Table S1.

#### Introduction Set

To introduce the water basin and the terms “floating” and “sinking,” the experimenter used a cork and a stone, respectively. As a visual reminder and to structure children’s explorations, two laminated sheets were used, one depicting a cork floating in a water basin and the word “float,” and the other depicting a stone at the bottom of a water basin and the word “sink” (adapted from Franse, 2007).

#### Exploration Sets

There were two exploration sets (Figure 1): one consisted of 10 common objects (used in non-systematic condition) and the other of 10 cubes (used in systematic condition). Both sets consisted of five floaters and five sinkers. Both sets had four items that were selected to be surprising for children based on the pilot study: two surprising floaters and two surprising sinkers. All of the systematic condition’s exploration set cubes were turquoise.

#### Test Set

The test set was the same for both conditions and was used to measure children’s predictions and explanations (Figure 1). It consisted of five common objects and five cubes. Of each set of five items (i.e., cubes or objects), two were selected to be easier and three were selected to be more difficult based on the pilot study. This resulted in four subsets: easy cubes (*n* = 2), easy common objects (the candy tin and the ceramic utensils rest; *n* = 2), difficult cubes (*n* = 3), and difficult common objects (the branch, bobby pin, and floor ball; *n* = 3). Of the easy items, one was a floater and one was a sinker. Of the difficult items, two were floaters and one was a sinker. All of the test set cubes were white.

### Procedure

#### Introduction

Children were tested individually. The experimenter first introduced the water basin and asked what would happen to the cork (or stone, order counterbalanced) and placed the item in the water basin once the child had responded. The experimenter asked the child what had happened and then provided the corresponding definition [i.e., “If something stays on the water, we (indeed) call that floating” and “If something goes to the bottom of the water basin, we (indeed) call that sinking”]. The experimenter pointed to the corresponding laminated sheet and placed the object on it. After this was performed for both example items, these were removed and the experimenter brought out the exploration set of that child’s condition.

#### Exploration

During the exploration session, the child was free to test the exploration set items in the water basin. The laminated float and sink sheets remained on the table for the child to use. Before starting, the child was encouraged to try to think about why some float and others sink. To further stimulate children to test all items and think about buoyancy, children were prompted by the experimenter at regular intervals. If after 30 s the child had not yet tried anything, the experimenter asked which item she wanted to put in the water first. After 3 min or if the child had indicated that she was finished before having tried all of the items, the experimenter asked if she had tried all of the items already. After 4 min or if the child indicated that she was finished, the experimenter asked if the child already knew why some float and some sink. After 5 min, the experimenter indicated that the time was up and removed the water basin and items.

#### Prediction

The experimenter placed the laminated float and sink sheets upright into two bins, creating a floating and a sinking bin. The experimenter then placed the test set items one by one in front of the child and asked whether the item would float or sink. If the child did not pick up the item, the experimenter instructed her to do so. Once the child made a prediction, she was instructed to place the item in the corresponding bin. This was repeated for all 10 items in a randomized order.

#### Explanation

The experimenter pulled the items belonging to one subset (i.e., the easy cubes, the easy common objects, the difficult cubes, or the difficult common objects) out of the bins, regardless of whether the child had sorted them correctly or not, and placed them on the table. The experimenter pointed to the items from the floating bin (if there were any) and the items from the sinking bin (if there were any) while saying, “You thought this/these would float and this/these would sink. Why do these float and do these sink?” After the child was done explaining, the experimenter asked, “Can you explain it some more?” This was repeated for each of the four subsets in a randomized order.

### Measures

#### Predictions

Children’s categorization of the test set items into the floating and the sinking bins was tallied in two ways: an accuracy score of incorrect (0) or correct (1) and a float–sink score of float (0) or sink (1).

#### Explanations

Children’s explanations were transcribed per item subset (i.e., easy cubes, easy objects, difficult cubes, and difficult objects), resulting in four sets of transcriptions per participant. The transcriptions were coded using an amended version of the coding scheme used by Franse et al. (under review). The final codes belonged to six nominal categories (1: other, 2: fact, 3: mass, 4: volume, 5: material, 6: mass and volume or scientific; see Supplementary Table S2). All applicable codes were allocated per subset. For example, a child’s explanation of the difficult objects could receive a 2, 4, and 5, for referring to a fact or experience, describing the item’s volume, and naming a material, respectively. Twenty percent (*n* = 40) of the transcriptions were re-coded by a second coder. The two coders had an overlap of 90.54%. Disparities were resolved on the basis of the coding scheme.

The data were subsequently reformatted for the analyses. A child received a binary absence (0) or presence (1) score for each explanation category, depending on whether their explanation had not or had included that category of explanation, respectively. This was done separately for each subset. In other words, per explanation category and per item subset, each child had a score of 0 if their explanation did not include this category and a score of 1 if their explanation did.

### Data Analysis

#### Predictions

To test whether the conditions affected the children’s predictions of the test set items’ buoyancy, a generalized linear mixed model (GLMM) for binomial data was performed with children’s prediction accuracies of the 10 items as the dependent variables and item type (cubes and objects) and condition (systematic vs. non-systematic) and their interaction as the independent variables. The random intercept effects of items and participants were included in the model. The GLMM was performed in the R package lme4 (R Core Team, 2017). However, to test how the conditions influenced the degree to which children’s predictions incorporated item features, we needed to capture the heterogeneity across children. For this, we used latent class analysis (LCA) techniques (Hickendorff et al., 2018) to identify patterns in children’s buoyancy predictions across the different items (i.e., across the five cubes and five common objects). The LCAs were performed separately for the cubes and the objects because of their different features and are explained below.

#### Cubes

To test how children’s predictions incorporated the relevant features (e.g., mass and volume) of the cubes, we used latent class regression analysis (LCRA) (Huang and Bandeen-Roche, 2004) following Franse et al. (under review). LCRAs have previously also been applied to cognitive development of other science concepts (Bouwmeester et al., 2004; Hofman et al., 2015). In the LCRAs, children’s buoyancy predictions of the five cubes are modeled as a function of the mass and the volume of each cube (plus an intercept). Different combinations of the cubes’ mass and volume in the buoyancy predictions can be captured by different classes, with each class being characterized by a regression equation. For example, one class might be characterized by a regression model that accurately integrates the mass and the volume of the cubes to predict buoyancy, and another class’ buoyancy predictions might best be modeled by a higher predictive load of mass than volume. Thus, each class identifies a solution strategy, namely, the way mass and volume were combined to decide items’ buoyancies, and hence the terms class and solution strategy can be used synonymously when interpreting the results. The logarithms of the cubes’ mass and volume were used as the predictors in the regression models because people’s perception of mass and volume operates on a logarithmic scale (Jones, 1986). Since children’s buoyancy predictions (i.e., the dependent variables) were scored as float (0) or sink (1), the models indicate the probability of a child predicting that a cube will sink.

One, two, and three class models were fit to the children’s float–sink predictions, including regression terms *b*_*mass*_, *b*_*volume*_, and the intercept, *a*. Akaike Information Criterion (AIC) and Bayesian Information Criterion (BIC) were used to select the optimal number of classes. Subsequent constrained models (with fewer regression terms) were compared using likelihood ratio tests based on Pearson’s χ^2^. Once the most parsimonious model was selected, the posterior probabilities were computed, indicating, for each child, the probability that the child belongs to each class based on their buoyancy predictions. The highest posterior probability was used to assign the child to a class.

#### Objects

Since the objects did not differ systematically in mass and volume, latent class analyses (McCutcheon, 1987) were used to detect underlying patterns in the dependent variables, namely, the accuracy of children’s buoyancy predictions across the five cubes. LCAs were fit with different numbers of classes, and models were compared using AIC, BIC, and bootstrapped model fit likelihood ratios (Hickendorff et al., 2018). The selected *n*-class model was subsequently made more parsimonious by constraining responses within classes and then between classes to be equal, freeing up degrees of freedom. LC(R)As were conducted using DepMixS4 in R (Visser and Speekenbrink, 2010) and MPlus (Muthén and Muthén, 2017).

#### Condition and Grade Effects

Following the “three-step approach” (Hickendorff et al., 2018) separate logistic regressions were carried out to test the effect of condition and grade on children’s cube class and object class memberships.

#### Explanations

Children’s explanations were also modeled using LCAs. In this manner, we could detect underlying patterns in the types of explanations children gave [i.e., the dependent variables were the absence (0) or presence (1) of each explanation category]. This is arguably more informative than examining the highest achieved explanation or the amount of explanation types given because it has the potential to reveal (combinations in) the types of features children’s explicit concepts of buoyancy rely on without defining these *a priori*. LCAs were again performed separately for the cubes and the objects, following the same procedure as for the predictions. The classes resulting from the LCAs differ in the degree to which class members were likely to use an explanation type per subset of items After the LCAs, separate logistic regressions were again performed on the cube and the object class memberships to test the effects of condition and grade.

#### Predictions vs. Explanations

To investigate whether there was a relation between children’s implicit and explicit responses, logistic regressions were carried out separately for the two item types. Children’s implicit class membership as well as grade were used to predict children’s explicit class membership.

## Results

### Buoyancy Predictions

**FIGURE 2:**
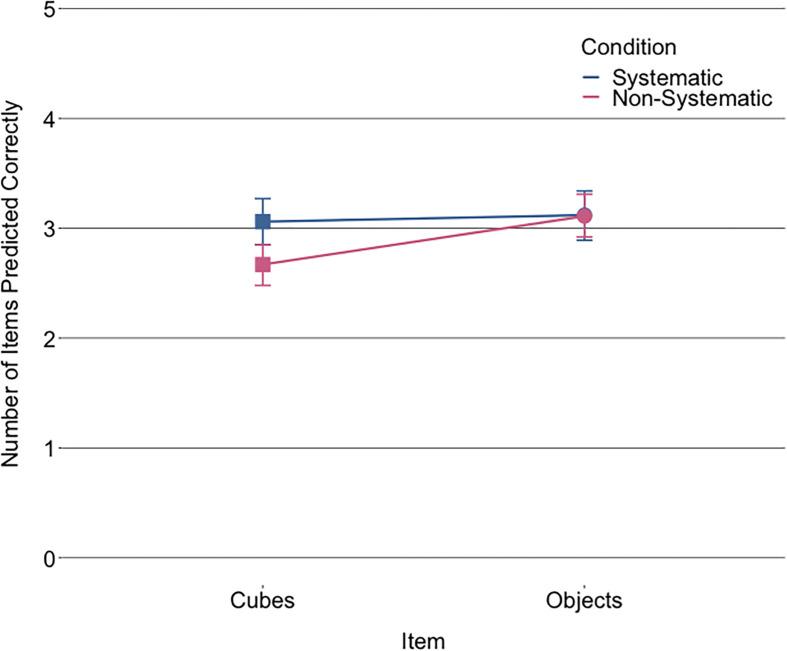
Mean number of accurate predictions of the cubes and objects at test per condition. Error bars denote 95% confidence intervals.

Children’s buoyancy prediction accuracy was compared across items between the two conditions using a GLMM (see Figure 2 for summed prediction accuracies per item type and Supplementary Figures S1, S2 for histograms). Table 2 shows the fixed and random effects of the GLMM (see Supplementary Table S3 for the correlations between the independent variables). The main effect of condition indicates that children in the non-systematic condition had a 0.35 lower odds of accurately predicting the buoyancy of an item than children in the systematic condition. The interaction between condition and item type, as displayed in Figure 2, indicates that children in the systematic condition were more accurate than children in the non-systematic condition, particularly on the cubes. This suggests that the systematic condition benefitted children’s prediction of the test set cubes while not greatly affecting children’s predictions of objects, whereas the non-systematic condition did not seem to benefit children’s object predictions relative to the systematic condition.

**TABLE 2:** Generalized linear mixed model statistics on the prediction accuracies.

	**Fixed effects**				**Random effects (SD)**	
	**Beta**	**SE**	***z*-value**	**Significance**	**Item**	**Participant**
Intercept	0.50	0.26	1.96	0.051	0.28 (0.53)	0.03 (0.17)
Condition	−0.35	0.14	−2.54	0.011		
Item type	0.03	0.36	0.08	0.93		
Condition * Item type	0.35	0.19	1.82	0.069		

**SE, standard error; SD, standard deviation.**

#### Cubes

To give an optimal description of individual differences in children’s buoyancy decisions about cubes, LCRA models with one to four classes were fit to children’s predictions of sinking (Table 3). The best-fitting, most parsimonious model was a three-class model, with two classes that included mass and volume predictors (“mass and volume” class: *b*_*mass*_ = −44.5, *b*_*volume*_ = 49.71, *a* = −22.19, 29% of the children; “MASS and volume class”: *b*_*mass*_ = −1.84, *b*_*volume*_ = 3.68, *a* = −5.83, 35% of the children) and one class that only included an intercept (“residual” class: *a* = −0.31, 36% of the children).

**TABLE 3:** Fit statistics for latent class regression models of cube predictions.

**Model**	**Classes**	**LR**	**Parameters**	**AIC**	**BIC**	***p* (LR)**
β_mass_ + β_vol_	1	−611.49	3	1,228.98	1,243.56	
β_mass_	1	−628.81	2	1,261.63	1,271.35	<0.001
β_vol_	1	−653.52	2	1,311.03	1,320.74	<0.001
1c: β_mass_ + β_vol_, 1c: *α*	2	−587.96	5	1,185.92	1,210.23	
1c: β_vol_,1c: *α*	2	−648.47	4	1,304.95	1,324.40	<0.001
1c: β_mass_, 1c: *α*	2	−612.68	4	1,233.37	1,252.82	<0.001
*2c: β_mass_ + β_vol_, 1c: *α*	3	−576.72	9	1,171.44	1,215.20	
1c: β_mass_ + β_vol_, 1c: β_mass_, 1c: *α*	3	−582.72	8	1,181.45	1,220.34	0.001
1c: β_mass_ + β_vol_, 1c: β_vol_, 1c: *α*	3	−582.52	8	1,181.03	1,219.93	0.001
3c: β_mass_ + β_vol_, 1c: *α*	4	−569.88	13	1,165.76	1,228.96	

**Model, regression terms per class; 1c, one class’ regression model included these terms; 2c, two classes’ regression models included these terms; 3c, three classes’ regression models included these terms; ^∗^, selected model; Classes, number of classes; LR, log likelihood ratio; AIC, Akaike Information Criterion; BIC, Bayesian Information Criterion; p (LR), p-value of likelihood ratio Pearson’s χ^2^.**

**FIGURE 3:**
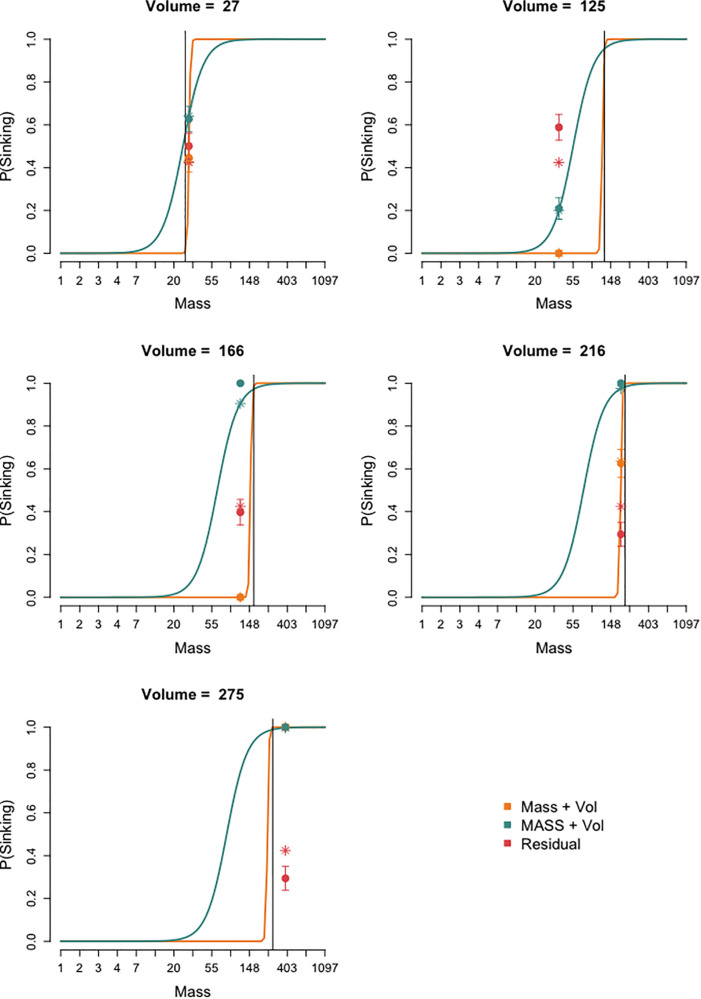
The three classes (depicted in different colors) found in the optimal latent class regression analysis of children’s predictions of cubes’ sinking. The probability of predicting that a cube will sink is depicted in each of the five graphs as a function of mass; each graph depicts the models for a different constant volume (i.e., the volumes of the five tested cubes: cube 11: *v* = 125, *m* = 37.5; cube 12: *v* = 166, *m* = 116.5; cube 13: *v* = 216, *m* = 194.5; cube 14: *v* = 27, *m* = 30; and cube 15: *v* = 275, *m* = 384.5). The black vertical line denotes the density of water (1 g/cm^3^); hence, cubes of constant volume with a mass to the left of this line would float and cubes of constant volume with a mass to the right of this line would sink. Each graph displays the modeled classes (curves as a function of mass and asterisks as a function of the predicted value for the mass of the tested cube) and the observed values (circles with standard errors). Classes “Mass and Volume” and “MASS and Volume” include all regression terms (hence, curves, and asterisks), while class “Residual” only included an intercept (hence, only asterisks). Note that the x-axis scale is logarithmic because logarithms of the cubes’ mass and volume were used.

The selected LCRA model is depicted in Figure 3, in which for each of the three classes (i.e., solution strategies), the probability of predicting that a cube will sink is plotted as a function of mass and volume. This is done separately in five graphs for the five constant volumes of the tested cubes (i.e., 125, 166, 216, 27, and 275 cm^3^), although note that the five graphs depict the same model. The black vertical line denotes the density of water (1 g/cm^3^), such that hypothetical cubes of constant volume with a mass to the left of this line would float and those of constant volume with a mass to the right of this line would sink. Take, for example, the volume of 125 cm^3^; the logistic curve of the “mass and volume” solution strategy closely aligns with the black density line, indicating that these children are modeled as correctly switching from predicting that a cube will float to predicting that it will sink when cubes have a density of around 1 g/cm^3^. This suggests that they quite accurately make a buoyancy decision based on mass and volume. For the tested cube of 125 cm^3^ and 37.5 g, this model therefore predicts that children will say that the cube will float (asterisk), which closely aligns with the observed buoyancy predictions (dot with error lines) of the children who were assigned to this class based on the posterior probabilities (see below). The “MASS and volume” logistic curve is less steep, indicating more uncertainty, and falls largely to the left of the density line. This implies that, at a lower mass, these children are already switching to predicting sinking, such that they seem to be over-compensating for mass. Indeed the model’s prediction of the probability that children will say that the tested cube (which floats) will sink is 0.2 (asterisk), overlapping with the observed buoyancy predictions for this cube (dot with error lines). Lastly, the “residual” class only has an intercept and therefore no regression slope. Overall, their observed (dot with error lines) and modeled (asterisk) buoyancy predictions are around chance, suggesting a guessing strategy.

**FIGURE 4:**
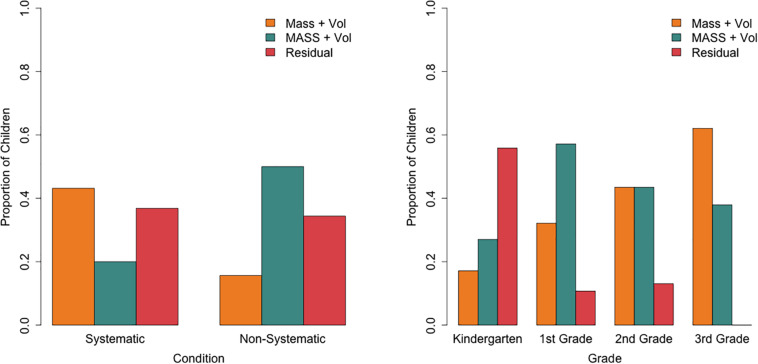
Proportion of children in each class per condition **(left)** and per grade **(right)**.

Children were assigned to one of the three classes based on the LCRA’s posterior probabilities, which indicate for each child the probability of that child belonging to each class based on the child’s actual buoyancy predictions. Next, a multinomial logistic regression revealed that this class membership could be predicted by condition and grade [Cox and Snell *R*^2^ = 0.37, χ^2^(6) = 89.17, *p*< 0.001; Figure 4]. Relative to the “MASS and volume” class, children in the systematic compared to the non-systematic condition had an odds of 7.60 (95%CI: 3.16–18.27) to belong to the “mass and volume” class, *b* = 2.03, *p* < 0.001. In other words, children in the systematic condition were more likely to belong to the “mass and volume” class than the “MASS and volume class,” while the opposite holds for children in the non-systematic condition. Children in the systematic condition compared to the non-systematic condition also had an odds of 9.97 (95%CI: 1.73–57.47) of belonging to the “residual” class relative to the “MASS and volume” class (*b* = 2.30, *p* = 0.01). As grade increases, the odds (0.06, 95% CI: 0.01–0.44) of belonging to the “residual” class relative to the “MASS and volume” class decreases (*b* = −2.81, *p* = 0.005). There was no significant interaction between condition and grade.

#### Objects

To give an optimal description of individual differences in children’s buoyancy decisions about objects, LCAs were performed on the prediction accuracy of the five test objects. One, two, and three class models were compared, leading to the selection of the two-class model (Table 4). This model was subsequently constrained. In the final model (LR = −583.80; *df* = 7; AIC = 1,181.60; BIC = 1,204.37), the log and the bobby pin were constrained to be equal within both classes, the floor ball and candy tin were constrained to be equal within one class, and the ceramic was constrained to be equal across classes, indicating that it did not distinguish class membership.

**TABLE 4:** Fit statistics for latent class models of common object predictions.

**Model**	**LR**	**Df**	**AIC**	**BIC**	**aBIC**	***p*^*a*^ (LR)**	***p*^a^ (PLR)**	**Entropy**
1 class	−609.91	5	1,229.81	1,246.07	1,230.24	0.00	na	
*2 class	−583.03	11	1,188.07	1,223.84	1,189.00	0.08	<0.001	0.56
3 class	−577.92	17	1,189.85	1,245.14	1,191.29	0.35	0.235	0.68

*, selected model; LR, log likelihood ratio; df, degrees of freedom; AIC, Akaike Information Criterion; BIC, Bayesian Information Criterion; aBIC, adjusted Bayesian Information Criterion; p (LR), p-value of model fit likelihood ratio Pearson’s χ^2^; p (PLR), p-value of parametric likelihood ratio for n-1 vs. n classes. ^*a*^Bootstrapped values.

**FIGURE 5:**
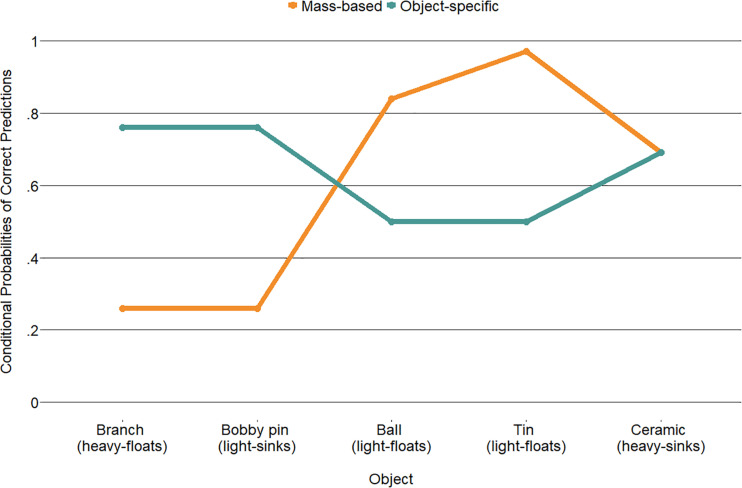
Latent class analysis probabilities of prediction accuracy for the two classes across the five objects.

Figure 5 displays the conditional probabilities of accurately predicting an object’s buoyancy for both classes. One class (54% of the children) performs poorly on two objects in which the mass is misleading (i.e., the branch floats but is relatively heavy and the bobby pin sinks but is relatively light) but performs better when predicting that relatively light items will float (i.e., the floor ball and the candy tin). The second class (46% of the children) performs well on the misleading objects (i.e., branch and bobby pin) but performs around chance on the light items (i.e., the ball that has holes and the candy tin with air inside that is made of metal). These two classes are tentatively interpreted as a class that seems to use primarily object mass to predict buoyancy and a class that makes object-specific predictions, respectively.

**FIGURE 6:**
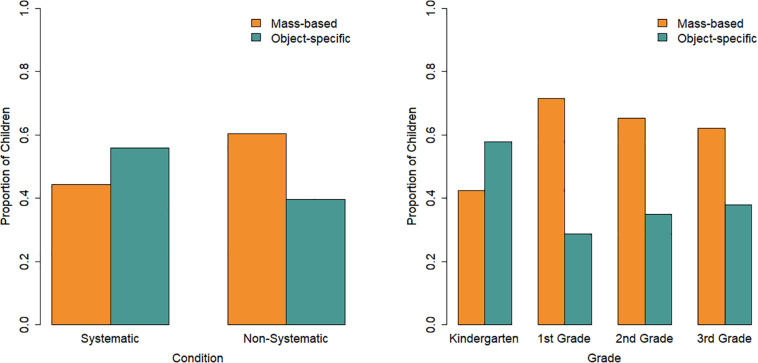
Proportion of children in each object prediction class per condition **(left)** and per grade **(right)**.

Limited evidence (Cox and Snell *R*^2^ = 0.07) was found for predictive effects of grade and condition on class membership as tested with a binary logistic regression (Figure 6). Children in the systematic condition had a higher odds (2.16, 95%CI: 1.18–3.94) than children in the non-systematic condition of belonging to the “object-specific” class relative to the “mass-based” class (*b* = 0.77, *p* = 0.012). As grade increases, the probability of belonging to the “object-specific” class also decreases (*b* = −0.57, *p* = 0.014). The interaction between condition and grade did not reach significance.

### Explanations

#### Cubes

The coded explanation categories were fit with LCAs to detect patterns in which types and combinations of buoyancy explanations children gave. Models with increasing numbers of classes were compared. Models with more than four classes did not result in stable solutions, leading to the selection of the four-class model (Supplementary Table S4). This model was subsequently constrained to make it more parsimonious. Constraints were applied across easy and difficult cubes, starting with the simpler categories (e.g., other, then fact) and subsequently to the more complex categories (e.g., mass and volume or scientific). Constraints were applied within one class and, subsequently, within increasing numbers of classes. Lastly, constraints across classes were tested.

In the final model (LR = −693.22; *df* = 30; AIC = 1,446.44; BIC = 1,544.01), the other explanation category was constrained across easy and difficult cube explanations for three classes. Fact was constrained to be equal between easy and difficult cube explanations for all classes, as was material which was even constrained across classes, indicating that it did not distinguish classes. Mass was only constrained to be equal across easy and difficult explanations for one class, while this could be done for volume in three classes. Finally, the mass and volume or scientific category was constrained in two classes.

**FIGURE 7:**
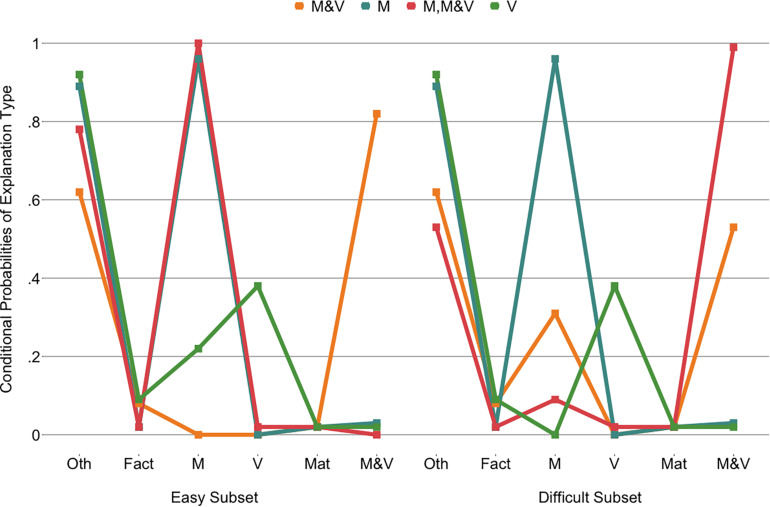
Latent class analysis probabilities of explanation types for the four classes across the two cube subsets. Oth, other; M, mass; V, volume; Mat, material; M&V, mass and volume, scientific.

The four classes (Figure 7) were interpreted based on the explanation categories class members were most likely to use. The “M” class (47% of the children) was found to have a high probability of appealing to the cubes’ mass for both the easy and the difficult cube sets, as well as providing explanations in the other category. The “V” class (25% of the children) primarily had explanations that fall into the other category but appealed to volume more than the other classes did. The “M, M&V” class (14% of the children) used mainly mass in their explanations for the easy subset and mass-and-volume or scientific explanations for the difficult subset. The “M&V” class (14% of the children) had an inversed pattern, although their explanations on the difficult subset were more mixed, including both mass and mass-and-volume or scientific explanations. Notably, all classes gave explanations falling in the other category, although this was most prominent for the “M” and “V” classes.

**FIGURE 8:**
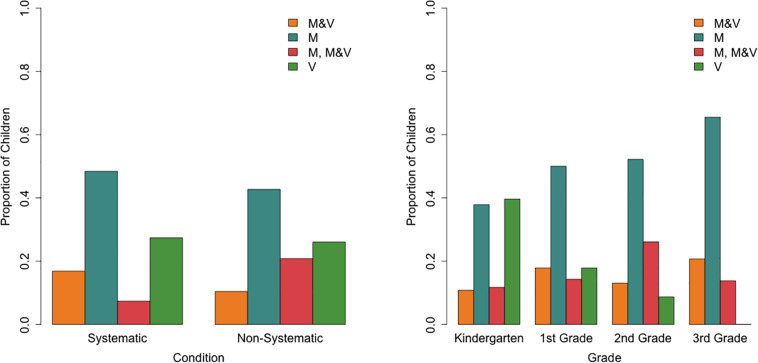
Proportion of children in each cube explanation class per condition **(left)** and per grade **(right)**.

A multinomial logistic regression with the “M” class as the reference class provided limited evidence for effects of condition and grade on class membership [Cox and Snell *R*^2^ = 0.19, χ^2^(9) = 40.39, *p*< 0.001; Figure 8]. Relative to the “M” class, the odds of belonging to the “M, M&V” class was lower for children in the systematic condition (0.32, 95%CI: 0.12–0.86) than in the non-systematic condition (*b* = -1.14, *p* = 0.023). As grade increased, the odds (0.37, 95%CI: 0.17–0.81) of belonging to the “V” class decreased relative to the “M” class (*b* = −1.00, *p* = 0.012). There was no significant interaction effect between grade and condition.

#### Objects

LCAs to detect patterns in children’s explanations of the objects were run in the same way as those for the cubes (Supplementary Table S5). Note that although the prediction accuracies (see Figure 5) did not necessarily reflect the *a priori* classification of the objects into easy and difficult subsets, explanations were asked for an entire subset at a time and could therefore not be analyzed separately per object. Models with more than five classes did not identify. The four-class model was selected as the BIC was lower than for the five-class model and for ease of interpretation because of the qualitative overlaps with the selected cube explanation model.

**FIGURE 9:**
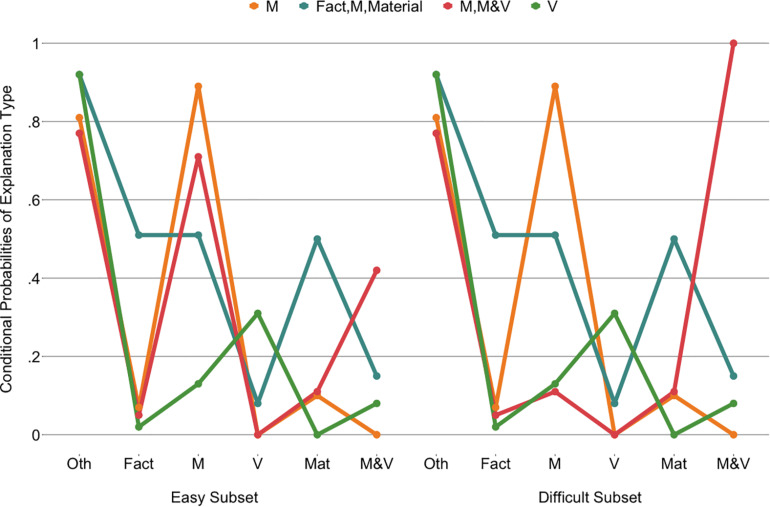
Latent class analysis probabilities of explanation types for the four classes across the two object subsets. Oth, other; M, mass; V, volume; Mat, material; M&V, mass and volume, scientific.

This model was constrained in the same theory-driven manner as the cube model. In the final model (LR = −903.89; *df* = 29; AIC = 1,865.79; BIC = 1,960.10), the other, fact, material, and volume categories were constrained across easy and difficult subsets for all classes. This was also the case for the mass and the mass and volume, scientific categories for three of the classes. No categories were constrained across classes. The classes of the final model (Figure 9) resembled those of the cube model: an “M” class (44% of the children), an “M, M&V” class (13% of the children), and a “V” class (18% of the children). The most notable difference was the presence of a class that appealed to facts and materials in addition to mass across easy and difficult objects, the “facts, M, material” class (25% of the children).

**FIGURE 10:**
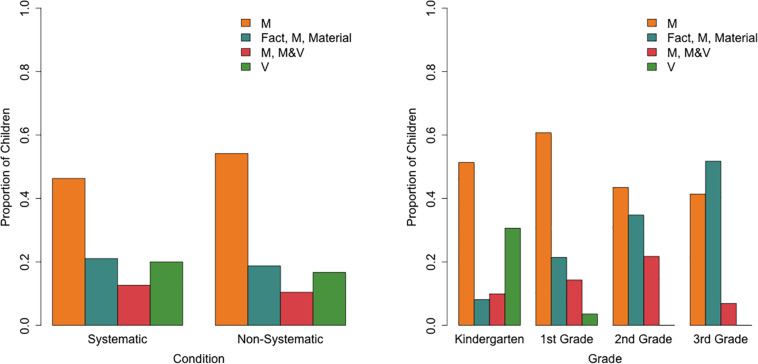
Proportion of children in each object explanation class per condition **(left)** and per grade **(right)**.

The multinomial logistic regression with the “M” class as the reference class revealed only one significant effect of grade on class membership [Cox and Snell *R*^2^ = 0.27, χ^2^(9) = 59.86, *p*< 0.001; Figure 10]. As grade increases, the odds (1.92, 95%CI: 1.19–3.08) of belonging to the “fact, M, material” class increases relative to the “M” class (*b* = 0.65, *p* = 0.007).

### Comparing Predictions and Explanations

**FIGURE 11:**
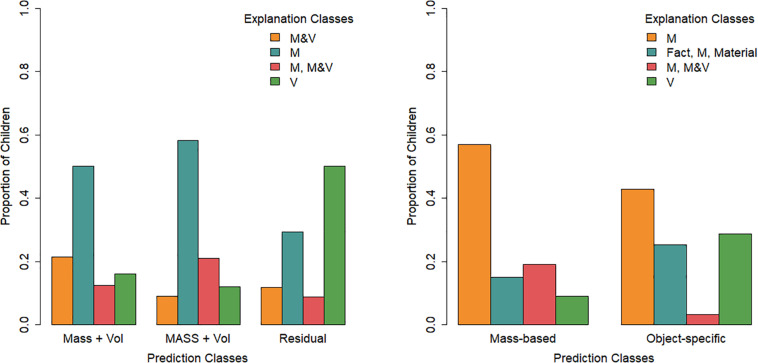
Proportion of children in each explanation class per prediction class for the cubes **(left)** and the objects **(right)**.

To test whether there was a relation between children’s predictions of cubes and their subsequent explanations (Figure 11) beyond grade effects, we performed a stepwise multinomial logistic regression in which grade and, subsequently, prediction class membership were added in separate steps. Adding prediction class membership did significantly improve the model [χ^2^(6) = 17.14, *p* = 0.009]. In this model [Cox and Snell = 0.23, χ^2^(9) = 48.51, *p*< 0.001], the children in the “MASS and volume” prediction class had a lower odds of belonging to the “V” explanation class relative to the “M” explanation class as compared to the “residual” prediction class. In other words, children who used the guessing solution strategy to predict the cubes’ buoyancy were more likely than the “MASS and volume” prediction class to belong to the “V” explanation class.

The same analysis was done for the object classes. The addition of the prediction classes improved the model of children’s explanation classes beyond the effects of grade [χ^2^(3) = 24.38, *p*<0.001]. Here [Cox and Snell = 0.34, χ^2^(6) = 80.28, *p*< 0.001] the prediction classes significantly predicted explanation class membership, with the “M” class as the reference class. Children in the “mass-based” prediction class had lower odds (0.23, 95%CI: 0.12–0.70, *b* = −1.25 *p* = 0.006) of belonging to the “fact, M, material” explanation class, higher odds (4.29, 95%CI: 1.17–15.77, *b* = 1.46, *p* = 0.028) of belonging to the “M, M&V” explanation class, and lower odds (0.35, 95%CI: 0.14–0.86, *b* = −1.05 *p* = 0.022) of belonging to the “V” explanation class as compared to the “object-specific” prediction class.

## Discussion

The current study compared the effect of children’s hands-on exploration of buoyancy using cubes (i.e., systematic condition) vs. common objects (i.e., non-systematic condition) on children’s subsequent predictions and explanations of new cubes’ and common objects’ buoyancies. Children’s responses were investigated using categorical latent variable models to account for individual differences.

### Predicting and Explaining Cubes

The latent class regression analyses of children’s cube buoyancy predictions identified three classes corresponding to three solution strategies. These three strategies replicate those identified by Franse et al. (under review). While in one class the children’s predictions were best modeled as guessing, two two-dimensional solution strategies were identified. The “mass and volume” solution strategy was characterized by a fairly accurate decision threshold, as indicated by a switch between predicting floating vs. sinking near a density of 1. This threshold was also modeled as being quite certain, as visible in the steepness of the decision curve (see Figure 3). The “MASS and volume” solution strategy was characterized as being less accurate as the threshold for predicting sinking was at a lower mass than would actually be the case for a given volume. Additionally, this solution strategy was also characterized by more uncertainty, seen in the more gradual slope from predicting floating to predicting sinking. Thus, both solution strategies made float–sink decisions based on the cubes’ relevant features (i.e., integrating mass and volume), although they differed in accuracy (i.e., how to relate the features to a decision; *cf*, Kuhn et al., 2008) and uncertainty (i.e., the strictness of the float–sink threshold).

Importantly, the proportion of children across the three classes differed between the two conditions. Children in the systematic condition were more likely to be in the “mass and volume” class than the “MASS and volume” class, while this was the other way around for those in the non-systematic condition. There was no evidence for an interaction between condition and grade, suggesting that the systematic condition was not more effective in one grade than another. These findings indicate that the 5 min of testing cubes in the water basin was effective in helping children to use a more accurate and certain solution strategy when faced with novel cubes.

Given that children in higher grades were also more likely to use the more advanced solution strategy, it is not the case that the systematic condition’s experience was *necessary* for using the “mass and volume” solution strategy. The effectivity of the systematic condition instead seems to lie in encouraging the switch to a more advanced integrative solution strategy. Since the two conditions differed in the proportion of children in one or the other integrative solution strategy but had similar proportions of children using the guessing strategy, it could further be postulated that this switch only occurs once a child already uses an integrative solution strategy but a less advanced one (e.g., the “MASS and volume” strategy; see also Siegler and Chen, 2008). This would need to be tested further by examining children’s strategy use before and after hands-on experience and by measuring solution strategy changes over a longer period of time.

Four classes of explanations were identified with latent class analyses (LCAs): “M&V,” “M,” “M, M&V,” and “V.” Grade effects revealed that children in higher grades were less likely to belong to the “V” class, which was characterized by mainly other and some mass and some volume category explanations, and more likely to belong to the “M” class, which only used other and mass categories. However, as expected given the limited duration and scope of the exploration phase, the effects of condition did not strongly carry over to children’s explanations of cubes. Only one condition effect was found; the “M, M&V” class membership was lower for systematic condition children than non-systematic condition children relative to the “M” class, yet the implications of this difference are unclear since several classes used mass and mass and volume explanations.

While by third grade all children were using a two-dimensional prediction solution strategy for the cubes, the majority of these children were explaining the cubes’ buoyancies primarily by referring to their masses. This suggests a discrepancy between predictions and explanations as found across past studies (Smith et al., 1985; Butts et al., 1993). Indeed the only significant predictive effect of prediction strategy on explanation strategy, beyond the effects of grade, indicated a relation between using the guessing prediction strategy and belonging to the “V” explanation class, which was characterized by primarily other category explanations. The absence of more clear relations suggests that it is difficult for children to verbalize their use of mass and volume or density (see also Dienes and Perner, 1999).

### Predicting and Explaining Common Objects

The accuracies of children’s predictions of the common objects were analyzed with LCAs to detect underlying patterns across predictions. Two classes were identified. In one, dubbed “mass-based,” the lighter objects had a higher probability of being accurately predicted to float, while a heavy floater and a light sinker were less accurately predicted. The second, “object-specific” class, had higher probabilities of correctly predicting the heavy floater and light sinker but were around chance at predicting the lighter objects. The “mass-based” class membership was slightly higher in the non-systematic than in the systematic condition.

Although it is not possible to determine which features children used to make these predictions, the most parsimonious interpretation would be that children based their explanations on a simple, one-dimensional strategy. The “mass-based” class responses could indeed be derived using the one-dimensional rule that heavy items sink and light items float. The “object-specific” class is trickier to interpret. These children could have used facts or object-specific past experiences to be more likely to correctly predict that a branch floats and a bobby pin sinks. The guessing behavior on the other items could stem from multiple sources of information; the ball was lightweight but had holes, reflecting the common idea that items with holes sink, and the tin was made of metal which typically sinks but was filled with air which typically causes objects to float (Hardy et al., 2006).

The relation between children’s common object predictions and explanations might help to further understand the prediction strategies. Four object explanation classes were identified: “M,” “fact, M, material,” “M, M&V,” and “V.” Whereas the majority of the children in the “mass-based” prediction class went on to belong to the “M” explanation class, the children in the “object-specific” prediction class were more distributed over the explanation classes. Notably, compared to the “mass-based” children, more “object-specific” children later belonged to the “fact, M, material” class or the “V” class which were both highly likely to also mention things in the other category. It could thus be that the “object-specific” prediction class is not so homogenous in how they came to the predictions but instead used several sources of information. However, due to the discrepancy between predictions and explanations in the literature (Rappolt-Schlichtmann et al., 2007; Franse et al., under review) using the explanations to interpret predictions is limited in its validity. Nonetheless, the predictive power of prediction class membership on explanation class membership does suggest that children’s predictions about common objects were related to how they went on to explain these objects’ buoyancy. As this was not the case for the cubes, this could suggest that the (multiple) features or facts used to discern objects’ buoyancies are more easily explicable than those used to predict cubes, perhaps because they are easier to detect or recall. Relatedly, the higher the grade, the more children belonged to the “fact, M, material” class, supporting the idea that many children go on to develop explanations of objects’ buoyancies based on multiple sources of information.

What is new in this investigation of buoyancy explanations is the open-ended manner of analysis. Past studies have assigned children to particular explanation types in a hierarchy from least to most advanced (Franse et al., under review) coded the complexity level of children’s explanations (Tenenbaum et al., 2004; Rappolt-Schlichtmann et al., 2007) or examined explanation profiles on the basis of answers to multiple choice buoyancy questions designed to reflect previously identified concepts (Schneider and Hardy, 2013; Edelsbrunner et al., 2018). Here coding all of the content of what children said and analyzing this with LCAs meant that we did not need *a priori* hierarchies or expected concepts, allowing us to discover relations in all that children say (see also van der Maas and Straatemeier, 2008). This method, for example, revealed that all explanation classes also mentioned things that fell into the other category, thus indicating that children do not have purely singular explicit concepts of buoyancy. Like any other explanation coding approaches, the findings here are still influenced by how the explanations were coded to begin with (e.g., what falls under other and what receives its own category). It is also important to note the explorative nature of this approach, and future work is needed to corroborate or extend these findings.

### Variation and Inquiry-Based Learning

This study indicates that providing children with the opportunity to explore the buoyancy of cubes stimulates the subsequent use of an advanced solution strategy when predicting whether novel cubes will float or sink. This benefit of using an item set that varies only in the relevant features is in line with the suggestions derived from category learning research (e.g., Sloutsky, 2010; Goldwater et al., 2018). In the systematic condition, the exploration cubes in effect presented children with dense categories; the only apparent features making the cubes either float or sink were the masses and volumes (hence, cubes’ densities were the defining feature between floaters and sinkers), thereby steering children to hone in on these features. In the non-systematic condition, however, multiple features might have been able to explain why some of the objects float and others sink, making it difficult for the children to identify mass and volume as the relevant features. Although the present findings require replication in future research, the successful findings of this very brief exploration session do hold promise for the use of variation and category denseness to design learning materials.

To understand how, this research can be placed in the context of inquiry-based learning. Across content domains, research indicates that well-guided inquiry-based learning is superior to explicit instruction and also that the presence and the quality of guidance is essential (Alfieri et al., 2011; Lazonder and Harmsen, 2016; Dobber et al., 2017). This is reiterated across several buoyancy studies, indicating that the more guidance the better and more prolonged the learning effects (Hardy et al., 2006; Rappolt-Schlichtmann et al., 2007; Hsin and Wu, 2011). Such guidance can be provided in different ways. For example, the present study used prompts to encourage children to test all of the items and to think about why they float or sink. Relatedly, the floating and the sinking sheets served as status overviews encouraging children to sort items, thus illustrating exploration progress. Perhaps the least explicit type of guidance is process constraints, which restrict the breadth of the learning task (Lazonder and Harmsen, 2016). The simplification of learning materials to vary only in density (i.e., the cubes) could be viewed as a process constraint as this restricted children’s exploration to the relevant features.

Whereas several studies have extensively investigated the longitudinal learning effects of mixed-method, long-term buoyancy interventions (Hardy et al., 2006; Schneider and Hardy, 2013; Leuchter et al., 2014; Edelsbrunner et al., 2018; Schalk et al., 2019), the aim of the current experimental study was simply to zoom in on the effect of item variation during hands-on exploration. Nevertheless, by placing the current findings in the context of inquiry-based learning, this study, like those classroom studies, emphasizes the importance of both considering fundamental learning processes in science education design and introducing science early (Leuchter et al., 2014). First, basing the design of inquiry-based learning guidance on a simple model of children’s category learning, as was done in this study, seems to provide an effective means of structuring the learning context. In future work, these ideas can be further worked out to provide an informed yet simple blueprint for designing inquiry-based learning founded on the (category) structure of the to-be learned concepts. This is important as teachers have been shown to not always be fully equipped with the relevant scientific knowledge or insights into children’s science learning (Rice, 2005; Leuchter et al., 2014). Second, the findings indicate that even in the first years of primary school, children can (implicitly) apply advanced multidimensional solution strategies when provided with the right environment, thereby echoing calls for starting science education early on using structured hands-on materials and learning environments (Leuchter et al., 2014).

In conclusion, this study investigated the effect of offering items that vary only in the relevant features (i.e., mass and volume) on children’s subsequent ability to predict and explain new items’ floating and sinking. In line with expectations, children who had had 5 min of hands-on experience with a set of cubes in the water basin were more accurate in predicting the buoyancy of new cubes than children who had used common objects to explore with. Moreover, these children were more likely to use a more advanced solution strategy that decided the buoyancy of an item based on the integration of its mass and volume than the children who had experimented with common objects. These findings suggest that inquiry-based learning designs should consider how to optimize the variation to facilitate learning.

## Data Availability Statement

The datasets generated for this study are available on request to the corresponding author.

## Ethics Statement

The studies involving human participants were reviewed and approved by the Faculteit der Sociale Wetenschappen Ethiek Commissie (ECPW) Instituut Pedagogische Wetenschappen. Written informed consent to participate in this study was provided by the participants’ legal guardian/next of kin.

## Author Contributions

JS, TS, RF, and MR conceived this project. JS and TS coordinated the data acquisition. JS and MR performed the data analyses. JS wrote the manuscript draft. TS, RF, and MR revised the manuscript draft. All authors contributed to the article and approved the submitted version.

## Conflict of Interest

The authors declare that the research was conducted in the absence of any commercial or financial relationships that could be construed as a potential conflict of interest.

## Footnotes

1 Note that Archimedes’ law of buoyancy provides a more general and an adequate way of thinking about floating and sinking, that is, “the relevant feature is the volume of the water to replace relative to the weight of the object” instead of “density.”

2 This was used as an exclusion criterion as a proxy of children’s engagement during the exploration session and to ensure that the included children understood the difference between floating and sinking.
