# Supplementary material for: Hands-On Exploration of Cubes’ Floating and Sinking Benefits Children’s Subsequent Buoyancy Predictions
Source: Front Psychol. 2020 Jul 21;11:1665. doi: 10.3389/fpsyg.2020.01665 (PMC7385235; doi:10.3389/fpsyg.2020.01665)
Supplement: Supplementary file 1 [file Data_Sheet_1.docx]

Supplementary Material

Table S1. Items per phase and item characteristics.

|  | **Condition** | **Item** | **Floats (F) or Sinks (S)** | **Material** | **Density (g/cm^3^)** | **Mass (g)** | **Rib length (cm)** | **Volume (cm^3^)** | **Previous Study Prediction Accuracy** | **Shape** |
| --- | --- | --- | --- | --- | --- | --- | --- | --- | --- | --- |
| Intro | Both | Wine cork | F | cork |  |  |  |  |  | cylinder |
|  |  | Stone | S | stone |  |  |  |  |  | stone |
| Exploration | Common Objects Condition | Ping pong ball | F | plastic |  |  |  |  | 0.90 | sphere |
|  |  | Wall plug | F | plastic |  |  |  |  | 0.67 | cone, hole |
|  |  | Boat | F | metal |  |  |  |  | 0.94 | boat |
|  |  | Pot coaster | F* | wood |  |  |  |  |  | square, holes |
|  |  | Bouncy ball | F* | rubber |  |  |  |  | 0.53 | sphere |
|  |  | Teaspoon | S | metal |  |  |  |  | 0.88 | spoon |
|  |  | 2 Euro Coin | S | metal |  |  |  |  | 0.91 (F) | disk |
|  |  | Marble | S | glass |  |  |  |  |  | sphere |
|  |  | Lava rock | S* | stone |  |  |  |  |  | stone |
|  |  | Sink sieve | S* | metal |  |  |  |  | 0.61 | disk, holes |
|  | Systematic Condition | Cube 1 | F |  | 0.4 | 10.80 | 3 | 27 | 0.87 | cube |
|  |  | Cube 2 | F |  | 0.4 | 25.60 | 4 | 64 |  | cube |
|  |  | Cube 3 | F |  | 0.8 | 6.40 | 2 | 8 |  | cube |
|  |  | Cube 4 | F* |  | 0.8 | 409.60 | 8 | 512 | 0.08 | cube |
|  |  | Cube 5 | F* |  | 0.9 | 247.16 | 6.5 | 274.63 |  | cube |
|  |  | Cube 6 | S* |  | 1.1 | 100.24 | 4.5 | 91.13 |  | cube |
|  |  | Cube 7 | S* |  | 1.1 | 17.19 | 2.5 | 15.63 |  | cube |
|  |  | Cube 8 | S |  | 1.6 | 200.00 | 5 | 125 | 0.92 | cube |
|  |  | Cube 9 | S |  | 1.6 | 68.60 | 3.5 | 42.88 | 0.89 | cube |
|  |  | Cube 10 | S |  | 1.8 | 299.48 | 5.5 | 166.38 |  | cube |
| Test | Both Conditions | Candy tin (glued shut) | F | metal |  |  |  |  | 0.96 | cylinder, air enclosed |
|  |  | Floor ball | F** | plastic |  |  |  |  | 0.46 (M) | sphere |
|  |  | Thick branch | F** | wood |  |  |  |  | 0.5 | cylinder |
|  |  | Chopsticks rest | S | ceramic |  |  |  |  | 1.0 | rectangular |
|  |  | Bobby pin | S** | metal |  |  |  |  | 0.56 | rectangular |
|  |  | Cube 11 | F |  | 0.3 | 37.5 | 5 | 125 | 0.87 | cube |
|  |  | Cube 12 | F** |  | 0.7 | 116.46 | 5.5 | 166.38 | 0.24 | cube |
|  |  | Cube 13 | F** |  | 0.9 | 194.4 | 6 | 216 |  | cube |
|  |  | Cube 14 | S** |  | 1.1 | 29.7 | 3 | 27 |  | cube |
|  |  | Cube 15 | S |  | 1.4 | 384.48 | 6.5 | 274.63 |  | cube |

Note: *Surprising items; **Difficult items; (F) = Franse et al. (under review) tested children between 4 and 12 years of age, (M) = internal Master’s thesis tested children between 8 and 12.

**Table S2.** Abbreviated coding scheme for explicit explanations of floating and sinking.

| **Code** | **Category** | **Subject of explanation** | **Examples** |
| --- | --- | --- | --- |
| 1 | Other | Don't know | "I don't know" |
|  |  | What does not fit elsewhere | "It’s not waterproof" |
|  |  | Irrelevant features | "It is soft" |
|  |  | Shape, form | "It is round" |
|  |  | Hole, air | "It has holes" |
| 2 | Fact | Correct or incorrect reference to fact or experience | "Rocks lie at the bottom of rivers" |
| 3 | Mass | Light, heavy | "This one is heavier" |
| 4 | Volume | Size, surface area | "It is really small" |
| 5 | Material | Correct or incorrect reference to material | "It is made out of metal" |
| 6 | Mass & Volume, Scientific | Mass and volume used during same explanation | "The rock is heavy. The ball is large." |
|  |  | Correct or incorrect incorporation of mass and volume | "It is heavy and small" |
|  |  | Correct or incorrect reference to scientific concept | "It is heavy but lighter than water" |

**
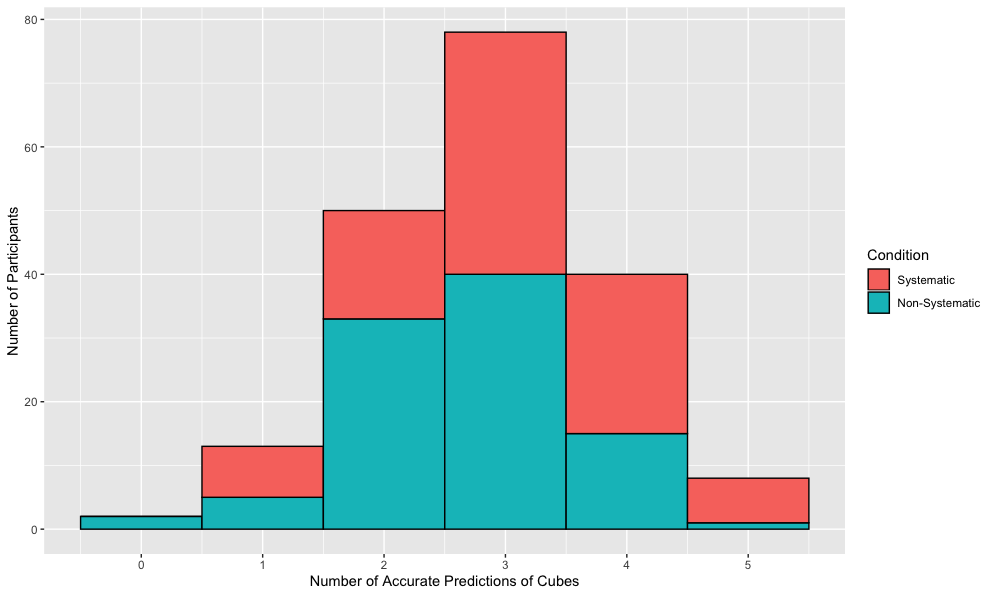
**

**Figure S1.** Histogram of Number of Accurately Predicted Cubes

**
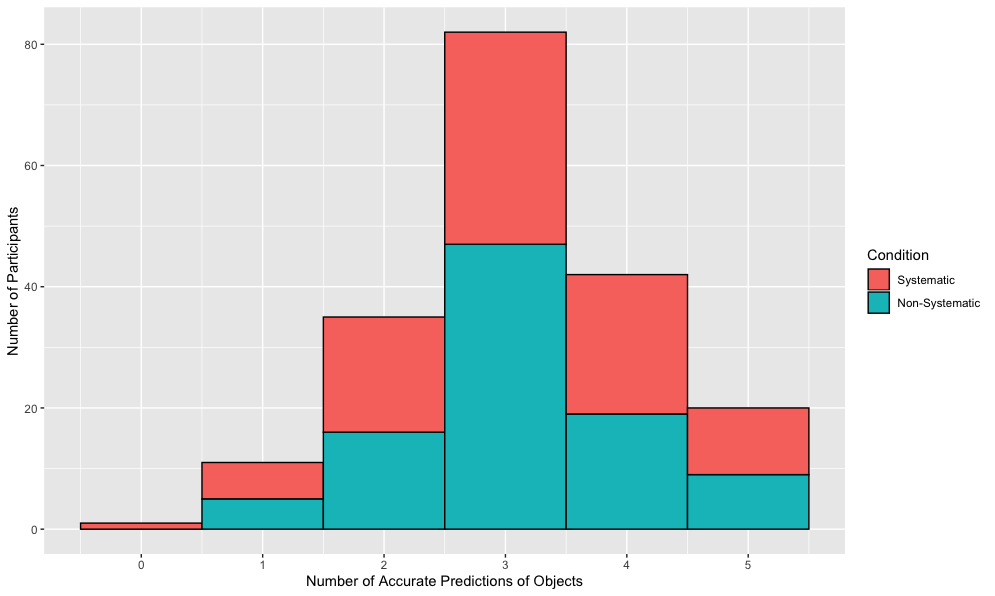
**

**Figure S2.** Histogram of Number of Accurately Predicted Objects

**Table S3.** Correlations of Fixed Effects

|  | Intercept | Condition | Item Type |
| --- | --- | --- | --- |
| Condition | -.28 |  |  |
| Item Type | -.71 | .19 |  |
| Condition*Item Type | .20 | -.70 | -.27 |

**Table S4.** Fit statistics for Latent Class Models of Cube Explanations

| **Model** | **LR** | **df** | **AIC** | **BIC** | **aBIC** | ***p*^a^(LR)** | ***p*^a^(PLR)** | **Entropy** |
| --- | --- | --- | --- | --- | --- | --- | --- | --- |
| 1 class | -838.59 | 12 | 1701.17 | 1740.20 | 1702.19 | 0.04 | na | na |
| 2 class | -757.95 | 25 | 1565.90 | 1647.21 | 1568.02 | 0.08 | < .001 | 1.00 |
| 3 class | -714.34 | 38 | 1504.67 | 1628.26 | 1507.89 | 0.08 | < .001 | 0.94 |
| *4 class | -683.62 | 51 | 1469.24 | 1635.11 | 1473.56 | 0.06 | < .001 | 0.95 |

*Note.* *, selected model*; LR*, log likelihood ratio; *df,* degrees of freedom; *AIC,* Akaike Information Criterion; *BIC,* Bayesian Information Criterion; *aBIC,* adjusted Bayesian Information Criterion; *p(LR)*, *p* value of model fit likelihood ratio Pearson's Chi^2^, p (PLR), p value of parametric likelihood ratio for n-1 versus n classes; ^a^bootstrapped values. Note that models with >4 classes did not result in stable solutions.

**Table S5.** Fit statistics for Latent Class Models of Object Explanations

| **Model** | **LR** | **df** | **AIC** | **BIC** | **aBIC** | ***p*^a^(LR)** | ***p*^a^(PLR)** | **Entropy** |
| --- | --- | --- | --- | --- | --- | --- | --- | --- |
| 1 class | -998.48 | 12 | 2020.96 | 2059.96 | 2021.97 | 0.03 | na | na |
| 2 class | -949.08 | 25 | 1948.16 | 2029.46 | 1950.27 | 0.02 | < .001 | 0.74 |
| 3 class | -920.15 | 38 | 1916.31 | 2039.89 | 1919.52 | 0.15 | < .001 | 0.72 |
| *4 class | -896.09 | 51 | 1894.18 | 2060.05 | 1898.50 | 0.07 | < .001 | 0.82 |
| 5 class | -877.82 | 64 | 1883.63 | 2091.78 | 1889.05 | 0.19 | 0.013 | 0.90 |

*Note.* *, selected model*; LR*, log likelihood ratio; *df,* degrees of freedom; *AIC,* Akaike Information Criterion; *BIC,* Bayesian Information Criterion; *aBIC,* adjusted Bayesian Information Criterion; *p(LR)*, *p* value of model fit likelihood ratio Pearson's Chi^2^, p (PLR), p value of parametric likelihood ratio for n-1 versus n classes; ^a^bootstrapped values. Note that models with >5 classes did not identifiy.
